# Community health information exposure and chronic disease preventive behavior among middle-aged and older adults an exploratory sequential mixed-methods study

**DOI:** 10.3389/fpubh.2026.1869256

**Published:** 2026-10-27

**Authors:** Jing Jing, Nuofan He, Wei Li

**Affiliations:** 1 College of Geography and Environment, Baoji University of Arts and Sciences, Baoji, China; 2 Shaanxi Key Laboratory of Disasters Monitoring and Mechanism Simulation, Baoji University of Arts and Sciences, Baoji, China; 3 Yangzhou University, Yangzhou, China

**Keywords:** chronic disease preventive behavior, community health information exposure, health literacy, mixed-methods research, preventive self-efficacy, risk perception

## Abstract

**Introduction:**

This study examined whether community health information exposure was associated with chronic disease preventive behavior among middle-aged and older adults, with particular attention to the chained mediating roles of risk perception and preventive self-efficacy and the moderating role of health literacy.

**Methods:**

An exploratory sequential mixed-methods design was adopted. In the qualitative phase, semi-structured interviews were conducted with 27 middle-aged and older community residents from six community health service centers in Sichuan and Chongqing, China. In the quantitative phase, 704 valid questionnaires were collected through site-based convenience sampling from eight community health service centers, three senior activity stations, and family-doctor-contracted populations. Confirmatory factor analysis, structural equation modeling, and bootstrap procedures were used to test the proposed model.

**Results and discussion:**

Community health information exposure was positively associated with chronic disease preventive behavior (β = 0.143, *p* < 0.01) and risk perception (β = 0.417, *p* < 0.001). Risk perception was further positively associated with preventive self-efficacy (β = 0.358, *p* < 0.001) and chronic disease preventive behavior (β = 0.119, *p* < 0.01). Preventive self-efficacy showed the strongest positive association with preventive behavior (β = 0.528, *p* < 0.001). Risk perception and preventive self-efficacy each showed significant indirect effects, and together formed a significant chained mediation path (indirect effect = 0.079, 95% CI [0.051, 0.115]). Health literacy significantly moderated the path from community health information exposure to risk perception (β = 0.154, *p* < 0.001) and was associated with a stronger chained indirect effect. Community health information exposure may contribute to chronic disease prevention partly through greater risk awareness and stronger preventive self-efficacy. Improving health literacy may help strengthen the translation of community health information into sustained preventive action, although longitudinal and intervention studies are still needed.

## 1 Introduction

As population aging continues, chronic non-communicable diseases have become a central public health problem affecting health status, quality of life, and primary healthcare resource allocation among middle-aged and older adults (1, 2). Hypertension, diabetes, dyslipidemia, obesity, and cardiovascular and cerebrovascular diseases not only increase disease burden at the individual level, but also intensify the long-term management pressure placed on community health services and family care (1, 2). Against this background, promoting stable and sustainable chronic disease preventive behavior through community-level health communication has become an important task in grassroots public health governance (3–6). In recent years, multiple forms of health communication have become increasingly common, including community health service centers, family-doctor contract follow-up, health lectures, public bulletin boards, WeChat-based health messages, and door-to-door reminders (3, 4). Opportunities for middle-aged and older residents to encounter health information have therefore expanded substantially. In theory, these communication channels should improve knowledge, increase awareness of chronic disease risk, and strengthen readiness for behavioral change (4, 7). In practice, however, a persistent gap remains between knowing and doing. Many residents can repeat the importance of salt reduction, routine health examinations, blood pressure monitoring, or adherence to medical advice, yet fail to sustain these behaviors in daily life, which suggests that exposure alone may be insufficient for sustained behavioral adoption (7–10). This persistent gap suggests that community health communication is not a simple process of information coverage (9, 11–13). Chronic disease preventive behavior is typically long-term, repetitive, and characterized by delayed benefits. Merely knowing what one should do is often insufficient to trigger sustained action. If health information is not interpreted as personally relevant risk and cannot be further transformed into the belief that “I am able to do this,” it may remain at the level of superficial cognition. Understanding how health information may be associated with behavior through specific psychological pathways is therefore central to explaining the observed effects of community-based chronic disease prevention communication. Although prior studies have reported associations between health information exposure and health behavior, three limitations remain (4, 14). First, many studies focus on whether individuals have encountered health information, but pay less attention to why such exposure is translated into durable behavioral change. Risk perception and self-efficacy have both been shown to matter for health behavior, yet they are commonly examined separately rather than integrated into a continuous behavioral formation framework (7, 15). Middle-aged and older adults differ substantially in health literacy, which may shape their ability to obtain, understand, evaluate, and use health information, thereby leading to different behavioral consequences under similar exposure conditions (16–20). To address these limitations, the present study proposes a chained pathway model in which community health information exposure is associated with chronic disease preventive behavior through risk perception and preventive self-efficacy. The study examines whether health literacy moderates the first segment of this pathway, namely the path from community health information exposure to risk perception. The study asks not only whether community health communication is associated with preventive behavior, but also how health information may relate to cognition and action beliefs among middle-aged and older adults, and whether this process differs by level of health literacy.

Compared with studies that treat health communication simply as information coverage or knowledge provision, the present study contributes in three ways. First, it integrates risk perception and preventive self-efficacy into a continuous explanatory framework that links community health information to chronic disease preventive behavior. It incorporates health literacy as a boundary condition and clarifies differentiated pathways through which information is transformed into psychological readiness and behavioral action. It adopts an exploratory sequential mixed-methods design to combine qualitative concept extraction with quantitative model testing in authentic community settings, thereby enhancing both contextual interpretability and statistical robustness.

## 2 Literature review and hypotheses

### 2.1 Community health information exposure and chronic disease preventive behavior among middle-aged and older adults

Chronic disease prevention and control has become a core task in community public health governance (1, 3). Compared with clinical treatment, early prevention, risk control, and behavior promotion at the community level are widely regarded as key strategies for reducing chronic disease burden and delaying functional decline (1, 3, 5, 6, 21). Community health service centers, family-doctor contract services, health education lectures, bulletin boards, WeChat-based messages, and home visits have all become important sources of routine health information for middle-aged and older adults (3, 4). From a health communication perspective, information exposure is usually treated as a necessary precondition for behavioral change. Individuals must first encounter relevant information before they can update knowledge, form risk cognitions, and generate behavioral responses. However, extensive public health practice shows that information exposure does not automatically equal behavior change. Many middle-aged and older adults repeatedly encounter advice such as “reduce salt and oil,” “exercise regularly,” and “monitor blood pressure,” but do not stably enact corresponding preventive behaviors. This indicates that the mechanism linking community health information to behavior is not simply one of “more exposure, more action,” but is likely shaped by both intervening psychological processes and individual capability conditions. For chronic disease prevention among middle-aged and older adults, the significance of information communication lies not only in knowledge transmission, but also in whether it is associated with problem awareness and behavioral readiness. On this basis, community health information exposure is conceptualized here as the starting-point variable in the behavioral formation process, consistent with broader health communication models in which message contact precedes risk appraisal and action readiness (4, 22). An important conceptual distinction concerns the boundary between information exposure, health literacy, and risk perception. In the present framework, community health information exposure refers primarily to contact with and engagement in community-delivered prevention messages, whereas health literacy represents a more general individual capacity to access, understand, evaluate, and use health information across situations. Similarly, perceived personal relevance of a message is conceptually distinct from risk perception: the former concerns whether the content of a message appears applicable to one's own circumstances, whereas the latter refers to a subjective appraisal of susceptibility, severity, and possible health consequences. Thus, information exposure is conceptualized as an upstream communication condition, health literacy as an information-processing capacity, and risk perception as a downstream threat appraisal. Nevertheless, because these constructs lie at adjacent stages of the information-processing sequence, some semantic proximity among particular questionnaire items is possible and is examined explicitly in the measurement and sensitivity analyses described below.

**H1**. Community health information exposure positively predicts chronic disease preventive behavior among middle-aged and older adults.

### 2.2 The mediating role of risk perception between information exposure and behavioral formation

Risk perception is a core variable in health behavior theory and generally refers to individuals' subjective judgments of susceptibility, severity, and possible consequences of a given health threat (7, 14). According to the Health Belief Model and Protection Motivation Theory, individuals are often willing to take preventive action only after they have developed a sense of personal risk (7, 14). Among middle-aged and older adults, many early signals of chronic disease are not highly salient, and risk awareness may therefore not arise spontaneously. Community health communication can repeatedly emphasize disease consequences, provide typical cases, and stress individual susceptibility, thereby transforming abstract health knowledge into personally relevant risk judgments. Qualitative materials in the present study similarly indicated that respondents became more likely to think “this could happen to me” after repeated community messages about hypertension, diabetes, and related complications. Previous studies often treat risk perception as an outcome variable in its own right and pay less attention to how it subsequently participates in behavioral formation. For long-term behaviors such as chronic disease prevention, however, risk perception may serve as a key psychological mediator through which information exposure influences action.

**H2**. Community health information exposure positively predicts risk perception.

**H3**. Risk perception positively predicts chronic disease preventive behavior.

**H4**. Risk perception mediates the relationship between community health information exposure and chronic disease preventive behavior.

### 2.3 The mediating role of preventive self-efficacy and its chained relationship with risk perception

Although risk perception helps activate disease vigilance, risk awareness alone is not enough to ensure that health behavior actually occurs. Extensive health promotion research shows that after recognizing health threats, individuals may still remain in a “knowing but not acting” state because they lack confidence, doubt whether they can persist, or are uncertain about the effectiveness of their actions (15, 23). Self-efficacy therefore represents an important complement to risk-based explanations of health behavior (15, 23). Preventive self-efficacy refers to an individual's belief that he or she can implement and sustain preventive actions, such as dietary control, regular physical activity, monitoring health indicators, or long-term adherence to medical advice. Among middle-aged and older adults, chronic disease prevention typically involves ongoing lifestyle adjustment, and both execution difficulty and persistence costs are relatively high. Knowing that chronic disease is risky is therefore insufficient; people must also believe that they are capable of acting. Qualitative findings in the present study showed that what truly motivated residents to undergo health examinations, regulate their diets, or monitor blood pressure was not merely knowledge of harm, but the feeling that they “could actually do it.” Preventive self-efficacy should therefore be treated as a proximal predictor of chronic disease preventive behavior rather than a peripheral variable. Risk perception and preventive self-efficacy are also not independent of each other. Health information may first increase the judgment “I am at risk,” and this heightened awareness may subsequently facilitate the belief “I can prevent this risk.” The relationship between the two thus forms a more behaviorally plausible chained pathway, in which perceived threat helps create motivational pressure and self-efficacy helps translate that pressure into feasible action (14, 15, 23).

**H5**. Community health information exposure positively predicts preventive self-efficacy.

**H6**. Risk perception positively predicts preventive self-efficacy.

**H7**. Preventive self-efficacy positively predicts chronic disease preventive behavior.

**H8**. Preventive self-efficacy mediates the relationship between community health information exposure and chronic disease preventive behavior.

**H9**. Risk perception and preventive self-efficacy form a significant chained mediation pathway in the relationship between community health information exposure and chronic disease preventive behavior.

### 2.4 The moderating role of health literacy as a boundary condition for community health communication

Although community health communication is formally addressed to all residents, individuals who are exposed to the same information do not necessarily show the same cognitive and behavioral responses. One important reason lies in differences in health literacy. Health literacy usually refers to the ability to obtain, understand, evaluate, and use health information for appropriate health decisions (4, 16). Among middle-aged and older adults, health literacy shapes not only whether health information is seen or heard, but whether its content is genuinely understood, recognized as personally relevant, and transformed into actionable behavioral strategies (16, 22). In the qualitative phase of this study, several respondents reported that they could not understand medical terminology or could not determine which suggestions were genuinely suitable for them. This indicates that information comprehension cannot be taken for granted in community health communication and may be a central source of effect heterogeneity. Middle-aged and older adults with higher health literacy are more likely to extract useful information from community campaigns, medical reminders, and health messages, and to transform that information into heightened personal risk perception. By contrast, those with lower health literacy may encounter the same information but fail to generate comparable levels of risk cognition. Health literacy may therefore strengthen the path from information exposure to risk perception and amplify the downstream chained mediation process, because residents with stronger information-processing skills are generally better able to interpret, evaluate, and use prevention messages in ways that inform personal health judgments (16–19).

**H10**. Health literacy positively moderates the relationship between community health information exposure and risk perception.

**H11**. Health literacy significantly moderates the chained mediation effect of “community health information exposure → risk perception → preventive self-efficacy → chronic disease preventive behavior.”

### 2.5 Research hypotheses and conceptual model

Based on the foregoing analysis, the present study constructs a public health behavioral model that includes direct effects, chained mediation effects, and a moderation effect (Table 1). Community health information exposure is treated as the independent variable, chronic disease preventive behavior as the outcome variable, risk perception and preventive self-efficacy as chained mediators, and health literacy as the moderator. The model is intended to clarify how community health communication promotes preventive behavior among middle-aged and older adults through cognitive activation and action-belief construction.

**Table 1:** Summary of research hypotheses.

Hypothesis	Content
H1	Community health information exposure positively predicts chronic disease preventive behavior among middle-aged and older adults.
H2	Community health information exposure positively predicts risk perception.
H3	Risk perception positively predicts chronic disease preventive behavior.
H4	Risk perception mediates the relationship between community health information exposure and chronic disease preventive behavior.
H5	Community health information exposure positively predicts preventive self-efficacy.
H6	Risk perception positively predicts preventive self-efficacy.
H7	Preventive self-efficacy positively predicts chronic disease preventive behavior.
H8	Preventive self-efficacy mediates the relationship between community health information exposure and chronic disease preventive behavior.
H9	Risk perception and preventive self-efficacy constitute a chained mediation path.
H10	Health literacy positively moderates the relationship between community health information exposure and risk perception.
H11	Health literacy significantly moderates the entire chained mediation process.

The proposed model contains three interrelated theoretical components. First, community health information exposure is specified as an exogenous starting condition that may be directly associated with chronic disease preventive behavior. This direct path is retained because repeated contact with community health messages may influence behavior through mechanisms not fully represented by the two proposed mediators, such as reminders, environmental cues, or access to community-based health services. The model therefore tests partial rather than complete mediation. Risk perception and preventive self-efficacy are specified as both individual mediators and sequential mediators. Risk perception represents the threat-appraisal stage through which general health information becomes personally relevant, whereas preventive self-efficacy represents the action-belief stage through which perceived risk is translated into confidence in initiating and sustaining preventive behavior. Accordingly, the model includes the paths from information exposure to risk perception, from risk perception to preventive self-efficacy, and from preventive self-efficacy to preventive behavior, while also retaining the two specific indirect pathways through risk perception and preventive self-efficacy separately. Health literacy is introduced as a first-stage boundary condition rather than as a general moderator of all structural paths. The theoretical rationale is that health literacy is most directly involved when individuals initially access, understand, evaluate, and personalize community health information. Residents with higher health literacy should therefore be more likely to transform similar levels of information exposure into stronger perceptions of personal chronic disease risk. Once risk perception has been formed, the subsequent relationships involving preventive self-efficacy and preventive behavior are modeled as common structural processes. In the structural equation model, community health information exposure and health literacy are treated as exogenous constructs, risk perception and preventive self-efficacy as sequential endogenous mediators, and chronic disease preventive behavior as the final endogenous outcome. The interaction between community health information exposure and health literacy is specified only for the path predicting risk perception. This formulation enables the SEM analysis to test the direct effect, the two specific indirect effects, the chained indirect effect, and the conditional chained indirect effect within one integrated theoretical framework.

## 3 Materials and methods

### 3.1 Research design and reporting framework

The study adopted an exploratory sequential mixed-methods design (24) (Figure 1). First, qualitative interviews were used to identify key experiences and relations through which community health information exposure was associated with chronic disease preventive behavior among middle-aged and older adults. Questionnaire measures were developed on the basis of qualitative findings, and the theoretical model was examined using quantitative data. This design is well suited to public health topics in which underlying pathways remain incompletely specified and must be clarified in authentic community settings (24). The study consisted of three phases. Phase 1 used qualitative inquiry to extract core concepts and construct the theoretical model. Phase 2 developed the measurement scales on the basis of qualitative findings and conducted a pilot test. Phase 3 used large-sample survey data and structural equation modeling to test direct effects, chained mediation effects, and moderation effects. To improve reporting completeness, the method section explicitly describes the sample sources, recruitment flow, inclusion and exclusion criteria, scale formation procedures, missing-data handling, and statistical strategy, with the qualitative phase reported in line with COREQ-oriented principles and the quantitative phase aligned with STROBE-oriented reporting expectations (25, 26). Because the quantitative phase relied on cross-sectional self-report data, all paths were interpreted as structurally consistent relations rather than strict causal effects. The mixed-methods connection was primarily a building design: qualitative themes were used to define construct boundaries, generate item domains, and refine wording before quantitative testing (24, 25).

**Figure 1:**
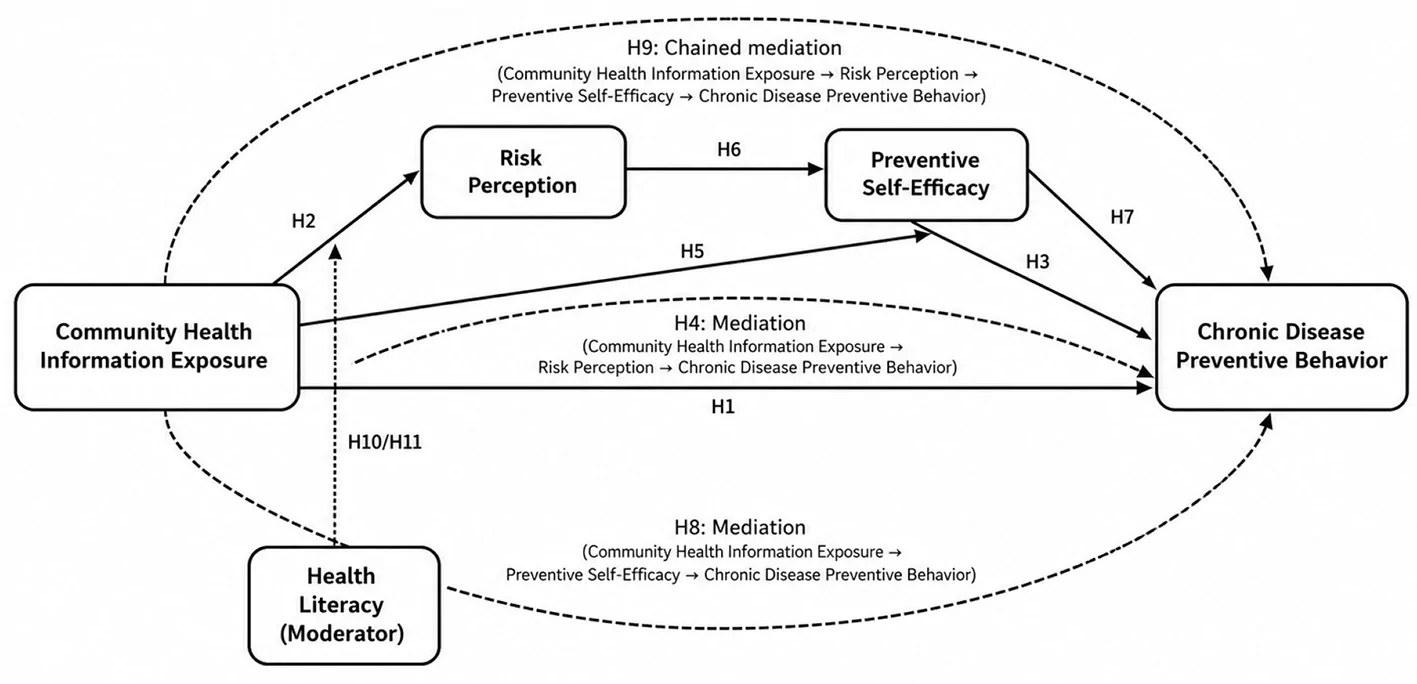
Theoretical structural equation model showing the direct effect of community health information exposure on chronic disease preventive behavior, the specific and chained mediating effects of risk perception and preventive self-efficacy, and the first-stage moderating effect of health literacy.

### 3.2 Qualitative phase: participants, interview guide, and analytic procedure

In the qualitative phase, 27 middle-aged and older residents were recruited from six community health service centers in Sichuan and Chongqing (Table 2). The sample comprised 12 men and 15 women, ranged in age from 52 to 76 years, and had a mean age of 64.9 years. Eighteen participants had been diagnosed with hypertension, diabetes, or dyslipidemia, and 21 reported a family history of chronic disease. Interviews focused on sources and channels of community health information, attention to chronic disease prevention messages, disease vigilance triggered by information, confidence in performing preventive actions, difficulties in understanding and applying health information, and reasons why greater health knowledge does not necessarily translate into sustained preventive behavior. Semi-structured one-to-one interviews were conducted, with each interview lasting approximately 40–65 min. Before each interview, participants were informed about the purpose of the study, the voluntary nature of participation, their right to decline participation or withdraw, the intended research use of the interview material, the procedures used to protect privacy and confidentiality, and the planned audio recording of the interview. Informed consent was obtained before the interview and audio recording commenced. All interviews were subsequently transcribed, and the interview material used for analysis and reporting was handled in de-identified form. The research team conducted thematic analysis through open coding, code clustering, and theme development. Four core themes were identified: “greater information visibility increases disease vigilance,” “risk awareness does not automatically generate action,” “behavioral initiation depends on operable confidence,” and “health literacy affects information absorption efficiency.” To strengthen the credibility of the qualitative findings, double coding, research memos, negotiated resolution of coding discrepancies, and saturation checks using later interviews were employed (25). The qualitative themes were subsequently used to inform the quantitative measurement domains. For community health information exposure, the qualitative material informed items concerning frequency and diversity of information contact, message visibility and attention, perceived comprehensibility, perceived personal applicability, and cognitive engagement with prevention information. Susceptibility, severity, and health-related concern informed the risk-perception items. Confidence in initiating, performing, and sustaining preventive actions informed the preventive self-efficacy items, whereas difficulties in accessing, understanding, evaluating, and applying health information informed the health-literacy items. Illustrative questionnaire items developed through this process included “I often receive chronic disease prevention information from my community,” “The health information I receive is closely related to my own health situation,” “Even when life becomes busy, I can still maintain key preventive behaviors,” and “I can tell whether community health information is reliable and useful.”

**Table 2:** Semi-structured interview guide.

Item	Core interview question
Q1	Through which community channels do you usually receive health information?
Q2	What type of chronic disease prevention information is most likely to attract your attention?
Q3	After receiving such information, does it change how you think about your own risk of chronic disease? If so, in what way?
Q4	Do you feel capable of sustaining changes in diet, exercise, and health monitoring?
Q5	Why do some people know a great deal about health but still fail to take action?
Q6	Is it difficult for you to understand and apply community health information? What kinds of difficulties do you encounter?

### 3.3 Quantitative phase: sample source, inclusion criteria, and survey procedure

In the quantitative phase, questionnaires were distributed through eight community health service centers, three senior activity stations, and family-doctor-contracted populations (Table 3). Recruitment followed site-based convenience sampling rather than probability sampling. The quantitative sample should therefore be interpreted as a community-based sample suitable for examining the proposed structural relationships rather than as a representative population survey. Sites were selected pragmatically to capture routine community public-health settings in which middle-aged and older residents commonly encounter chronic disease prevention information, including clinic-based health education, senior activity-station outreach, and family-doctor follow-up. Within each setting, trained investigators approached potentially eligible residents on site or during regularly scheduled community activities, briefly introduced the study, screened eligibility, and invited participation on a voluntary basis. Residents from family-doctor-contracted populations were approached during follow-up contacts or community service sessions rather than through random household sampling. Participants were eligible if they were aged 50 years or older, had lived in the community for more than 6 months, had encountered at least one source of community health information during the previous 6 months, and were able to complete the questionnaire independently. Before questionnaire administration, potential participants were informed about the purpose and procedures of the study, the voluntary nature of participation, their right to decline participation or withdraw, the intended research use of the survey data, and the procedures used to protect privacy and confidentiality. Informed consent was obtained before questionnaire completion. Questionnaires were completed on paper, and trained investigators were available to clarify procedural or wording questions but were instructed not to suggest or influence substantive responses. A total of 820 questionnaires were distributed and 781 were returned, corresponding to a return rate of 95.2%. Data-quality screening excluded questionnaires with missing key items, clearly abnormal completion times, repeated identical response patterns accompanied by logical contradictions, or responses indicating no community health information exposure during the previous 6 months and therefore failure to satisfy the exposure-related eligibility criterion. After these exclusions, 704 questionnaires were retained for analysis. The retained questionnaires represented 90.1% of the returned questionnaires and 85.9% of all questionnaires distributed. The final analytic sample included 302 men (42.9%) and 402 women (57.1%), with a mean age of 63.8 years (SD = 7.12). Among the respondents, 46.3% had been diagnosed with at least one chronic disease, 52.8% reported a family history of chronic disease, and 71.6% had received community health education during the previous year. The investigation covered three routine community-service scenarios community health service centers, senior activity stations, and family-doctor-contracted populations to reduce reliance on a single recruitment setting. Of the 77 returned questionnaires that were excluded, most were removed because of missing key variables or clear response-pattern problems. The project archive did not retain a reliable site-by-site refusal log; consequently, a formal non-response analysis could not be conducted. The analytic file did not retain complete recruitment-site identifiers for all participants, precluding formal multilevel modeling of site-level clustering. These issues are considered when interpreting the representativeness of the sample and the precision of the estimated associations.

**Table 3:** Basic characteristics of the quantitative sample.

Characteristic	Category	Percentage/value
Gender	Male/Female	42.9%/57.1%
Mean age	–	63.8 years (SD = 7.12)
Diagnosed with at least one chronic disease	–	46.3%
Family history of chronic disease	–	52.8%
Received community health education in the past year	–	71.6%
Questionnaires distributed/returned/retained	–	820/781/704

### 3.4 Measures and operational definitions

Five core constructs were measured: community health information exposure, risk perception, preventive self-efficacy, health literacy, and chronic disease preventive behavior (Figure 2). Questionnaire development followed a theme-to-domain-to-item procedure derived from the qualitative phase. Recurrent interview themes were translated into conceptually defined measurement domains, including information visibility and engagement, perceived susceptibility and severity, confidence in performing and sustaining preventive actions, difficulties in accessing and evaluating health information, and routine chronic disease preventive practices. Draft items were written in plain language suitable for middle-aged and older adults. Two public health researchers and one community-health practitioner reviewed the initial item pool for conceptual relevance, wording clarity, redundancy, and appropriateness for community settings. A pilot administration was subsequently conducted to identify ambiguous or overly technical wording and to reduce respondent burden before the final field version was established. The supplementary material reports the item wording, construct assignment, substantive domains, response format, coding procedure, and construct-scoring approach. Representative items are retained in the main text to illustrate the operational meaning of each construct. Community health information exposure was assessed using seven items reflecting the extent to which respondents encountered and engaged with chronic disease prevention information delivered through community-based channels. The items covered frequency of contact, diversity of information sources, visibility and attention to prevention messages, perceived comprehensibility of the messages encountered, perceived personal applicability, and subsequent cognitive engagement with prevention information. Representative items included “I often receive chronic disease prevention information from my community” and “The health information I receive is closely related to my own health situation.” Higher scores represented greater exposure to and engagement with community-based chronic disease prevention information. Because some components of the broader exposure measure are conceptually adjacent to health literacy and risk perception, their conceptual boundaries require clarification. Perceived comprehensibility in the exposure measure referred to the perceived clarity of specific community messages encountered, whereas health literacy represented a more general perceived capacity to obtain, understand, evaluate, and apply health information across situations. Similarly, perceived applicability or self-relevance referred to whether a message appeared pertinent to the respondent's circumstances, whereas risk perception referred to subjective judgments of disease susceptibility, severity, and health-related concern. The potential for residual overlap among these adjacent constructs was therefore examined through the discriminant-validity analyses described in Section 3.5 and the narrower-exposure sensitivity analysis reported in Section 4.6.

**Figure 2:**
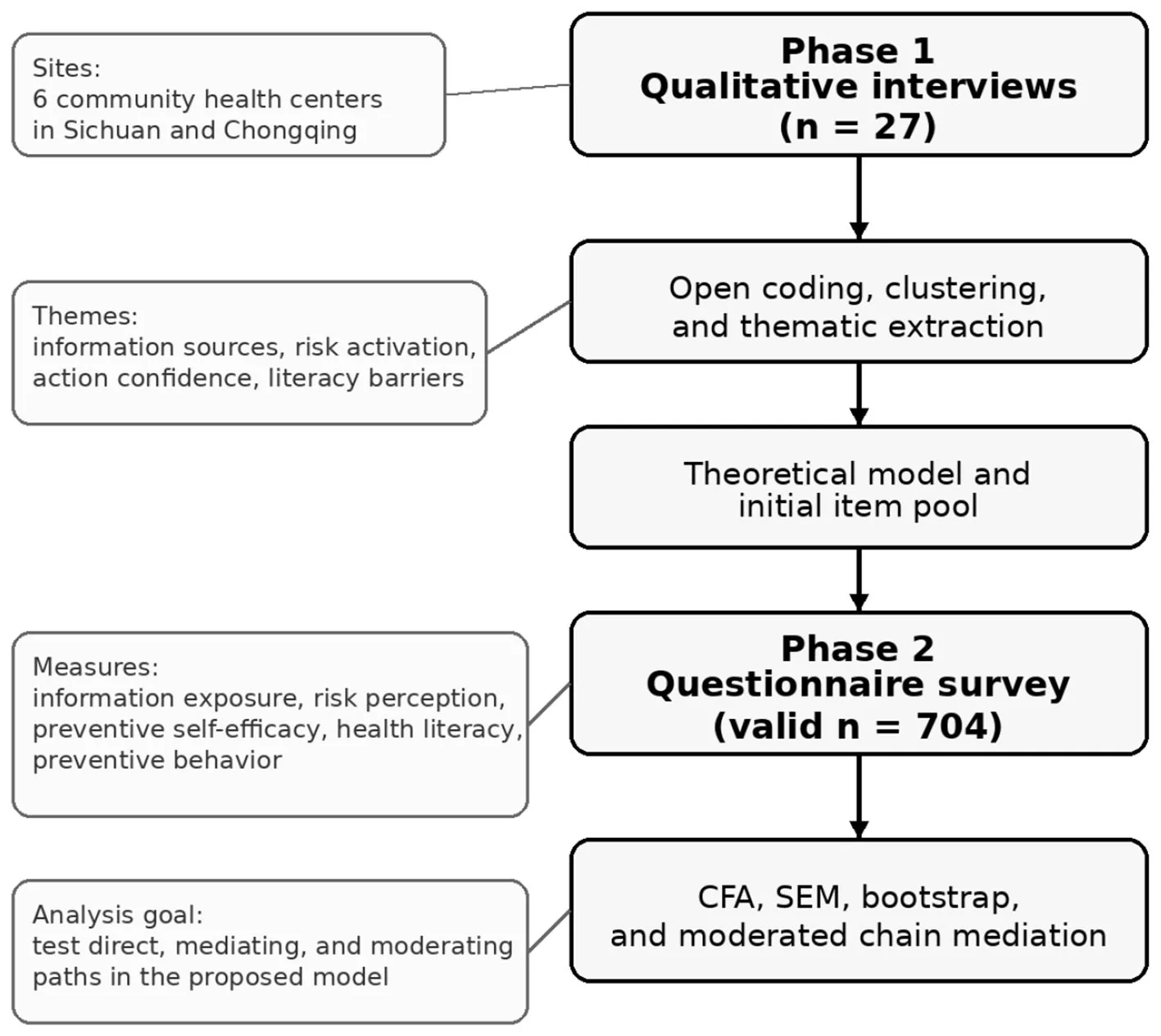
Exploratory sequential mixed-methods design.

Risk perception was assessed using six items covering perceived susceptibility to chronic disease, perceived seriousness of potential consequences, and concern about future health risks. A representative item was “If I do not manage my health well, I am likely to develop or worsen a chronic disease.” Higher scores indicated stronger perceived personal risk and greater perceived seriousness of chronic disease. Preventive self-efficacy was assessed using seven items addressing confidence in dietary control, regular physical activity, health monitoring, adherence to preventive recommendations, and persistence in preventive behavior when daily routines were disrupted. A representative item was “Even when life becomes busy, I can still maintain key preventive behaviors.” Higher scores indicated stronger confidence in initiating and sustaining chronic disease preventive actions. Health literacy was assessed using six items reflecting the perceived ability to obtain, understand, evaluate, and apply health information. A representative item was “I can tell whether community health information is reliable and useful.” Higher scores indicated a stronger perceived capacity to access, interpret, appraise, and use health-related information in everyday decision making. Chronic disease preventive behavior was assessed using eight items covering routine physical examinations, blood pressure or blood glucose monitoring when appropriate, dietary control, regular physical activity, adherence to medical or preventive recommendations, and smoking- and alcohol-related risk control. A representative item was “I regularly monitor indicators such as blood pressure or blood glucose when needed.” Higher scores indicated more frequent and consistent self-reported engagement in chronic disease preventive practices. All 34 measurement items were rated on a five-point Likert scale ranging from 1 (*strongly disagree*) to 5 (*strongly agree*). No items were reverse-coded, and higher item scores consistently represented higher levels of the corresponding construct. For descriptive reporting and construction of the observed moderation term described in Section 3.5, construct scores were calculated as the arithmetic means of their corresponding items. For CFA and the primary structural measurement component, the individual items were specified as indicators of their respective latent constructs. Although chronic disease preventive behavior referred to specific health practices, it was measured through self-report rather than objective clinical records or device-based observations. The behavioral findings should therefore be interpreted as self-reported preventive practices and as evidence statistically consistent with the proposed structural relationships rather than as objectively verified behavioral performance.

### 3.5 Reliability, validity, and statistical analysis strategy

All statistical analyses were conducted using IBM SPSS Statistics version 26.0 and IBM SPSS Amos version 26.0. SPSS was used for data screening, descriptive statistics, internal-consistency reliability analysis, bivariate correlation analysis, construction of the interaction term, and supplementary analyses. Confirmatory factor analysis (CFA) and structural equation modeling (SEM) were conducted in Amos using maximum likelihood (ML) estimation. All analyses were based on the 704 valid questionnaires retained after the data-cleaning procedures described in Section 3.3. Internal consistency was evaluated using Cronbach's α. The Kaiser–Meyer–Olkin (KMO) statistic and Bartlett's test of sphericity were used to assess the suitability of the item data for factor analysis. The five-factor measurement model was subsequently evaluated using CFA. Convergent validity was assessed using standardized factor loadings, composite reliability (CR), and average variance extracted (AVE). Overall model fit was evaluated using the chi-square-to-degrees-of-freedom ratio (χ^2^/*df*), comparative fit index (CFI), Tucker–Lewis index (TLI), incremental fit index (IFI), root mean square error of approximation (RMSEA), and standardized root mean square residual (SRMR). Given the conceptual proximity among community health information exposure, health literacy, and risk perception, discriminant validity was examined beyond inspection of the inter-construct correlations. Heterotrait–monotrait ratios (HTMT) were calculated for the five constructs, with particular attention to the community health information exposure–health literacy and community health information exposure–risk perception pairs. HTMT values below the prespecified threshold of 0.85 were interpreted as supporting discriminant validity. As an additional check, the hypothesized five-factor measurement model was compared with alternative measurement models in which conceptually adjacent constructs were combined. Better fit of the five-factor model relative to these alternative specifications was interpreted as additional evidence that the constructs were empirically distinguishable.

Because all core constructs were measured by self-report at a single time point, procedural precautions were used to reduce potential common method bias. These included anonymous questionnaire administration, standardized instructions, and separation of the major construct blocks in the questionnaire. Residual common method bias was nevertheless treated as a methodological limitation rather than assumed to have been eliminated by these procedural measures. The structural component of the model specified community health information exposure as the exogenous predictor, risk perception and preventive self-efficacy as sequential endogenous mediators, and chronic disease preventive behavior as the final endogenous outcome. Health literacy was specified as a moderator of the first-stage path from community health information exposure to risk perception. For notational clarity, let *X* denote community health information exposure, *M*_1_ risk perception, *M*_2_ preventive self-efficacy, *W* health literacy, and *Y* chronic disease preventive behavior.

Before introducing the interaction term, the serial-mediation component can be represented by the following structural Equations 1–3:

$$\begin{array}{ll} M_{1} & =\alpha_{1}+a_{1}X+\varepsilon_{1}, \end{array}$$ (1)

$$\begin{array}{ll} M_{2} & =\alpha_{2}+a_{2}X+d_{21}M_{1}+\varepsilon_{2}, \end{array}$$ (2)

$$\begin{array}{ll} Y & =\alpha_{3}+c^{\prime }X+b_{1}M_{1}+b_{2}M_{2}+\varepsilon_{3}. \end{array}$$ (3)

In this specification, *c*′ denotes the direct association between community health information exposure and chronic disease preventive behavior. Three specific indirect pathways were evaluated: the indirect effect through risk perception (*a*_1_*b*_1_), the indirect effect through preventive self-efficacy (*a*_2_*b*_2_), and the chained indirect effect through risk perception and preventive self-efficacy (*a*_1_*d*_21_*b*_2_). The total indirect effect was defined as the sum of these three indirect components, and the total effect was defined as the direct effect plus the total indirect effect.

For the moderation analysis, construct-level scores for community health information exposure and health literacy were calculated as the means of their respective items solely for the purpose of constructing the interaction term. To reduce nonessential multicollinearity and facilitate interpretation of the lower-order effects, both variables were mean-centered (Equation 4):

$$\begin{array}{l} X_{c}=X-\bar{X},\;W_{c}=W-\bar{W}. \end{array}$$ (4)

The interaction term was then constructed in SPSS as Equation 5

$$\begin{array}{l} XW=X_{c}\times W_{c}. \end{array}$$ (5)

The centered lower-order terms (*X*_*c*_ and *W*_*c*_) and their product term (*XW*) were retained simultaneously in the moderation model. The interaction term was entered into Amos as an observed exogenous product term predicting risk perception. Accordingly, the moderation component should be interpreted as an observed first-stage interaction embedded within the broader five-factor serial-mediation SEM rather than as a latent-variable interaction model. The first-stage moderation equation was specified as Equation 6

$$\begin{array}{l} M_{1}=\alpha_{1}+a_{1}X_{c}+\gamma_{1}W_{c}+a_{3}\left(X_{c}W_{c}\right)+\varepsilon_{1}, \end{array}$$ (6)

where *a*_3_ represents the interaction between community health information exposure and health literacy. A statistically significant *a*_3_ indicates that the association between community health information exposure and risk perception varies according to the level of health literacy.

Significant interactions were interpreted using simple-slope analysis at one standard deviation below and one standard deviation above the mean of health literacy. Given the observed mean of 3.39 and standard deviation of 0.73, these probing values corresponded to approximately 2.66 (−1 SD) and 4.12 (+1 SD) on the original health-literacy scale. Mediation and moderated mediation were evaluated using a non-parametric case-resampling bootstrap procedure with 5,000 resamples. In each bootstrap iteration, cases were sampled with replacement from the analytic sample, and the relevant structural parameters and indirect effects were re-estimated. The specific indirect effects, chained indirect effect, total indirect effect, conditional chained indirect effects, and index of moderated mediation were summarized using 95% bootstrap confidence intervals. An indirect or moderated indirect effect was considered statistically supported when its 95% bootstrap confidence interval did not include zero.

Because health literacy moderated the *X*→*M*_1_ path, the conditional chained indirect effect was defined as Equation 7

$$\begin{array}{l} IE\left(W\right)=\left(a_{1}+a_{3}W_{c}\right)d_{21}b_{2}. \end{array}$$ (7)

The index of moderated mediation was defined as Equation 8

$$\begin{array}{l} \text{IMM}=a_{3}d_{21}b_{2}. \end{array}$$ (8)

This index quantifies the linear change in the chained indirect effect associated with a one-unit increase in health literacy. Evidence of moderated mediation was inferred when the 95% bootstrap confidence interval for the index excluded zero. Two sets of supplementary robustness analyses were conducted. First, age, gender, family history of chronic disease, and receipt of community health education during the previous year were included as covariates to examine whether adjustment for plausible demographic and health-related confounders materially altered the principal structural relationships. Because the primary community health information exposure measure contained items related to perceived comprehensibility and personal relevance, a sensitivity analysis was conducted using a narrower four-item exposure specification. This specification retained CHE1–CHE4, which primarily represented message-contact frequency, source diversity, message visibility, and attention, and excluded CHE5–CHE7, which contained greater conceptual proximity to information comprehension, personal relevance, or subsequent cognitive engagement. The reliability of the narrower measure, its correlations with health literacy and risk perception, the principal structural paths, model-fit indices, and the chained indirect effect were re-evaluated. This narrower specification was treated as a robustness analysis rather than as a replacement for the primary seven-item measure. Unless otherwise specified, all statistical tests were two-sided, with *p* < 0.05 considered statistically significant.

## 4 Results

### 4.1 Sample characteristics and integrated qualitative findings

In the formal sample, women slightly outnumbered men, the mean age was approximately 64 years, and more than 40% of respondents had at least one chronic disease diagnosis or a family history of chronic disease (Table 4). This structure indicates that the sample captured a population with relatively high chronic disease risk exposure and therefore matched the problem addressed in the present study, although it should not be treated as population-representative. Qualitative findings indicated that community health information first functioned by activating disease vigilance, but whether such vigilance was associated with preventive behavior depended on whether residents developed sufficient confidence to act.

**Table 4:** Reliability and validity indicators of the scales.

Variable	Items	Cronbach's α	KMO	CR	AVE
Community health information exposure	7	0.894	0.876	0.903	0.571
Risk perception	6	0.908	0.891	0.916	0.647
Preventive self-efficacy	7	0.923	0.913	0.929	0.653
Health literacy	6	0.887	0.868	0.895	0.592
Chronic disease preventive behavior	8	0.931	0.927	0.938	0.656

### 4.2 Measurement model: reliability, validity, and correlations

All five scales demonstrated satisfactory internal consistency. Cronbach's α values ranged from 0.887 to 0.931, while KMO values ranged from 0.868 to 0.927. Bartlett's tests of sphericity were significant for all constructs (*p* < 0.001), indicating that the item data were suitable for factor analysis. Confirmatory factor analysis further showed standardized factor loadings ranging from 0.65 to 0.88. Composite reliability (CR) values ranged from 0.895 to 0.938, and average variance extracted (AVE) values ranged from 0.571 to 0.656. Taken together, these results supported satisfactory internal consistency and convergent validity of the five-construct measurement model.

Because community health information exposure, health literacy, and risk perception represent conceptually adjacent stages of the information-processing pathway, discriminant validity was examined using the heterotrait–monotrait ratio (HTMT) in addition to the inter-construct correlations. As shown in Table 5, all HTMT values were below the prespecified threshold of 0.85. In particular, the HTMT value between community health information exposure and risk perception was 0.62, whereas that between community health information exposure and health literacy was 0.37. The highest HTMT value was 0.74 for preventive self-efficacy and chronic disease preventive behavior, which also remained below the threshold. These results suggest that the five constructs were empirically distinguishable despite their theoretical proximity.

**Table 5:** Correlations and heterotrait–monotrait ratios among the main variables.

Variable	1	2	3	4	5	6
1. Community health information exposure	–	0.62	0.49	0.37	0.42	
2. Risk perception	0.46^***^	–	0.52	0.28	0.38	
3. Preventive self-efficacy	0.39^***^	0.43^***^	–	0.41	0.74	
4. Health literacy	0.29^***^	0.21^***^	0.34^***^	–	0.46	
5. Chronic disease preventive behavior	0.34^***^	0.31^***^	0.61^***^	0.37^***^	–	
6. Age	0.08^*^	0.11^**^	0.06	0.04	0.09^*^	–

Pearson correlation coefficients are reported below the diagonal. HTMT values for the five focal latent constructs are reported above the diagonal. HTMT was not calculated for age because age was treated as an observed covariate rather than a latent construct. All HTMT values were below the prespecified threshold of 0.85. ^*^p < 0.05, ^**^p < 0.01, ^***^p < 0.001.

The discriminant validity of the measurement model was further examined by comparing the hypothesized five-factor model with alternative four-factor models in which conceptually adjacent constructs were combined. The hypothesized five-factor model showed satisfactory fit (χ^2^/*df* = 1.786, CFI = 0.968, TLI = 0.962, IFI = 0.968, RMSEA = 0.034, SRMR = 0.039). When community health information exposure and health literacy were combined into a single factor, model fit deteriorated (χ^2^/*df* = 2.438, CFI = 0.932, TLI = 0.925, IFI = 0.933, RMSEA = 0.045, SRMR = 0.058). A second four-factor model combining community health information exposure and risk perception also showed poorer fit (χ^2^/*df* = 2.711, CFI = 0.918, TLI = 0.910, IFI = 0.919, RMSEA = 0.049, SRMR = 0.064). The superior fit of the five-factor model provided additional support for treating community health information exposure, health literacy, and risk perception as distinct constructs.

Bivariate correlations were consistent with the proposed structural relationships. Community health information exposure was positively correlated with risk perception (*r* = 0.46, *p* < 0.001), preventive self-efficacy (*r* = 0.39, *p* < 0.001), health literacy (*r* = 0.29, *p* < 0.001), and chronic disease preventive behavior (*r* = 0.34, *p* < 0.001). Risk perception was positively correlated with preventive self-efficacy (*r* = 0.43, *p* < 0.001) and preventive behavior (*r* = 0.31, *p* < 0.001). The strongest correlation among the focal constructs was observed between preventive self-efficacy and chronic disease preventive behavior (*r* = 0.61, *p* < 0.001). Although these correlations supported the expected directions of association, none was sufficiently large to suggest that the focal constructs were statistically redundant.

### 4.3 Structural model fit and direct effects

The structural analysis proceeded in two steps. The serial-mediation structural model without the interaction term was estimated to examine the direct and mediating pathways among community health information exposure, risk perception, preventive self-efficacy, and chronic disease preventive behavior. This base structural model demonstrated satisfactory fit to the data (χ^2^/*df* = 1.924, CFI = 0.961, TLI = 0.955, IFI = 0.961, RMSEA = 0.036, and SRMR = 0.043). The observed interaction term between mean-centered community health information exposure and health literacy, constructed as described in Section 3.5, was added to the first-stage equation predicting risk perception. The resulting moderated structural model also showed satisfactory fit (χ^2^/*df* = 1.951, CFI = 0.958, TLI = 0.952, IFI = 0.958, RMSEA = 0.037, and SRMR = 0.045). The modest change in the fit indices after inclusion of the interaction term indicated that the moderation component could be incorporated without materially reducing the overall adequacy of the structural model. The principal structural coefficients are reported in Table 6. Community health information exposure was positively associated with chronic disease preventive behavior (β = 0.143, *p* < 0.01), risk perception (β = 0.417, *p* < 0.001), and preventive self-efficacy (β = 0.119, *p* < 0.01). Risk perception was positively associated with preventive self-efficacy (β = 0.358, *p* < 0.001) and chronic disease preventive behavior (β = 0.119, *p* < 0.01). Preventive self-efficacy showed the strongest association with chronic disease preventive behavior (β = 0.528, *p* < 0.001). In the moderated model, health literacy retained a positive lower-order association with risk perception (β = 0.104, *p* = 0.004). More importantly, the interaction between community health information exposure and health literacy was positively associated with risk perception (β = 0.154, *p* < 0.001), indicating that the positive association between information exposure and risk perception became stronger as health literacy increased. The interaction effect is examined in greater detail through simple-slope and moderated-mediation analyses in Section 4.5.

**Table 6:** Main structural path coefficients.

Path	Standardized β	*p*	Interpretation
Community health information exposure → Chronic disease preventive behavior	0.143	< 0.01	Greater information exposure was associated with more frequent preventive behavior.
Community health information exposure → Risk perception	0.417	< 0.001	Greater information exposure was associated with stronger perceived chronic disease risk.
Community health information exposure → Preventive self-efficacy	0.119	< 0.01	Greater information exposure was associated with stronger confidence in preventive action.
Risk perception → Preventive self-efficacy	0.358	< 0.001	Greater risk perception was associated with stronger preventive self-efficacy.
Risk perception → Chronic disease preventive behavior	0.119	< 0.01	Greater risk perception was associated with more frequent preventive behavior.
Preventive self-efficacy → Chronic disease preventive behavior	0.528	< 0.001	Preventive self-efficacy showed the strongest proximal association with preventive behavior.
Health literacy → Risk perception	0.104	0.004	Higher health literacy was positively associated with risk perception after adjustment for information exposure and the interaction term.
Community health information exposure × Health literacy → Risk perception	0.154	< 0.001	The association between information exposure and risk perception became stronger as health literacy increased.

Standardized coefficients for the direct and mediation-related paths are reported together with the lower-order health-literacy effect and the first-stage interaction effect. The interaction term was constructed from mean-centered community health information exposure and health literacy scores as described in Section 3.5.

### 4.4 Mediation analysis

The bootstrap procedure described in Section 3.5 was applied to the two specific indirect effects, the chained indirect effect, the total indirect effect, and the total effect (Table 7). Based on 5,000 resamples, the specific indirect effect of community health information exposure on chronic disease preventive behavior through risk perception was 0.050, with a 95% bootstrap confidence interval of [0.024, 0.083]. Because this interval did not include zero, the indirect effect through risk perception was statistically supported. The specific indirect effect through preventive self-efficacy was also statistically supported, with an effect of 0.063 and a 95% bootstrap confidence interval of [0.036, 0.098] (Table 8). The chained indirect effect through risk perception and preventive self-efficacy was 0.079, with a 95% bootstrap confidence interval of [0.051, 0.115]. Thus, the sequence of community health information exposure → risk perception → preventive self-efficacy → chronic disease preventive behavior was statistically supported. The total indirect effect was 0.192, with a 95% bootstrap confidence interval of [0.142, 0.252], and the total effect was 0.335, with a 95% bootstrap confidence interval of [0.274, 0.396]. Because none of the confidence intervals for the indirect effects included zero, the findings were statistically consistent with both specific and chained mediation. These results suggest that the association between community health information exposure and preventive behavior is not explained by information coverage alone, but is also associated with greater risk vigilance and stronger confidence in preventive action.

**Table 7:** Indirect effects.

Effect path	Effect type	Effect value	95% CI
Information exposure → Risk perception → Preventive behavior	Specific indirect effect	0.050	[0.024, 0.083]
Information exposure → Preventive self-efficacy → Preventive behavior	Specific indirect effect	0.063	[0.036, 0.098]
Information exposure → Risk perception → Preventive self-efficacy → Preventive behavior	Chained indirect effect	0.079	[0.051, 0.115]
Total indirect effect	Bootstrap	0.192	[0.142, 0.252]
Total effect	Bootstrap	0.335	[0.274, 0.396]

**Table 8:** Conditional chained indirect effects and index of moderated mediation.

Effect	Estimate	95% bootstrap CI	Interpretation
Conditional indirect effect at high health literacy (+1 SD)	0.104	[0.069, 0.146]	Stronger chained indirect association
Conditional indirect effect at low health literacy (−1 SD)	0.051	[0.027, 0.082]	Weaker chained indirect association
High–low difference	0.053	[0.019, 0.091]	Conditional indirect effects differ across the two probing values
Index of moderated mediation	0.036	[0.013, 0.062]	The chained indirect effect increases as health literacy increases

High and low health literacy correspond to one standard deviation above and below the sample mean, respectively. With *M* = 3.39 and *SD* = 0.73, these probing values correspond approximately to 4.12 and 2.66 on the original scale. Estimates were evaluated using 5,000 nonparametric bootstrap resamples. An indirect effect or index of moderated mediation was considered statistically supported when its 95% bootstrap confidence interval did not include zero.

### 4.5 Moderation and moderated chained mediation

Health literacy significantly moderated the association between community health information exposure and risk perception (Table 9). After mean-centering the two lower-order variables and entering their product term into the model, the interaction coefficient was positive and statistically significant (β = 0.154, *p* < 0.001, 95% CI [0.089, 0.219]). This result indicates that the positive association between community health information exposure and risk perception became stronger as health literacy increased. Simple-slope analysis was conducted at one standard deviation above and below the mean of health literacy. At a relatively high level of health literacy (+1 SD; approximately 4.12 on the original scale), the slope of community health information exposure predicting risk perception was 0.48 (*SE* = 0.05, *t* = 9.60, *p* < 0.001). At a relatively low level of health literacy (−1 SD; approximately 2.66), the corresponding slope was 0.26 (*SE* = 0.06, *t* = 4.33, *p* < 0.001). The difference between the two simple slopes was 0.22 (*SE* = 0.07, *t* = 3.14, *p* = 0.002), indicating a stronger information-exposure–risk-perception association among respondents with higher health literacy. The first-stage moderation was then incorporated into the chained indirect pathway (Table 10). Using 5,000 non-parametric bootstrap resamples, the conditional chained indirect effect of community health information exposure on chronic disease preventive behavior through risk perception and preventive self-efficacy was 0.104 (95% bootstrap CI [0.069, 0.146]) at high health literacy and 0.051 (95% bootstrap CI [0.027, 0.082]) at low health literacy. The difference between these conditional indirect effects was 0.053 (95% bootstrap CI [0.019, 0.091]), indicating that the chained indirect association was stronger at higher levels of health literacy. In addition to comparing conditional indirect effects, the index of moderated mediation was used as the formal inferential test of whether the chained indirect effect varied linearly with health literacy. The index was 0.036, with a 95% bootstrap confidence interval of [0.013, 0.062]. Because this interval did not include zero, the moderated chained mediation effect was statistically supported. Thus, higher health literacy was associated not only with a stronger first-stage relationship between community health information exposure and risk perception, but also with a stronger downstream indirect pathway through risk perception and preventive self-efficacy (Table 11).

**Table 9:** Simple slope analysis of the moderating effect of health literacy.

Health literacy level	Simple slope β	SE	*t*	*p*
High health literacy (+1 SD)	0.48	0.05	9.60	< 0.001
Low health literacy (−1 SD)	0.26	0.06	4.33	< 0.001
Slope difference	0.22	0.07	3.14	0.002

**Table 10:** Descriptive statistics of the main variables.

Variable	M	SD	Skewness	Kurtosis
Community health information exposure	3.58	0.67	−0.31	0.42
Risk perception	3.46	0.70	–0.18	0.11
Preventive self-efficacy	3.61	0.66	–0.27	0.33
Health literacy	3.39	0.73	–0.14	–0.08
Chronic disease preventive behavior	3.44	0.71	–0.22	0.20

**Table 11:** Comparison of model fit indices.

Model	χ^2^/*df*	CFI	TLI	IFI	RMSEA	SRMR
Measurement model	1.786	0.968	0.962	0.968	0.034	0.039
Structural model	1.924	0.961	0.955	0.961	0.036	0.043
Model with moderation term	1.951	0.958	0.952	0.958	0.037	0.045

### 4.6 Supplementary analyses: subgroup differences and robustness checks

Supplementary analyses were conducted to examine whether the principal findings were sensitive to respondent characteristics and to the operational definition of community health information exposure. These analyses were treated as robustness checks rather than as substitutes for the primary structural model. Subgroup comparisons were conducted according to gender, chronic disease diagnosis, and receipt of community health education during the previous year. As shown in Table 12, gender differences in risk perception, preventive self-efficacy, and chronic disease preventive behavior were small and were not statistically significant. By contrast, respondents who had been diagnosed with at least one chronic disease reported higher levels of risk perception, preventive self-efficacy, and preventive behavior than respondents without a diagnosed chronic disease. Respondents who had received community health education during the previous year also reported higher scores on all three outcomes than those who had not received such education. The principal structural relationships were further re-estimated after adjusting for age, gender, family history of chronic disease, and receipt of community health education during the previous year. The signs and statistical interpretations of the main structural coefficients remained unchanged after covariate adjustment. These findings suggest that the principal pattern of associations was not attributable solely to the measured demographic and health-related characteristics. Because the study was observational and cross-sectional, however, these adjusted analyses should be interpreted as robustness checks rather than as evidence that all potential confounding was eliminated.

**Table 12:** Group differences in risk perception, preventive self-efficacy, and preventive behavior.

Grouping variable	Category	Risk perception	Preventive self-efficacy	Preventive behavior	Between-group tests
Gender	Male	3.42 ± 0.71	3.57 ± 0.68	3.41 ± 0.73	RP: *t* = −1.12, *p* = 0.262; PSE: *t* = −1.18, *p* = 0.238; PB: *t* = −1.10, *p* = 0.272
	Female	3.48 ± 0.69	3.63 ± 0.65	3.47 ± 0.70	
Chronic disease status	Diagnosed	3.61 ± 0.66	3.69 ± 0.63	3.56 ± 0.68	RP: *t* = 5.19, *p* < 0.001; PSE: *t* = 3.01, *p* = 0.003; PB: *t* = 4.32, *p* < 0.001
	Not diagnosed	3.34 ± 0.72	3.54 ± 0.69	3.33 ± 0.73	
Community health education in past year	Received	3.53 ± 0.68	3.71 ± 0.64	3.54 ± 0.67	RP: *t* = 4.30, *p* < 0.001; PSE: *t* = 5.20, *p* < 0.001; PB: *t* = 5.69, *p* < 0.001
	Not received	3.27 ± 0.74	3.41 ± 0.71	3.19 ± 0.76	

Values are means ± standard deviations. RP, risk perception; PSE, preventive self-efficacy; PB, chronic disease preventive behavior. Between-group statistics are reported separately for each outcome.

#### 4.6.1 Sensitivity analysis using a narrower information-exposure specification

To evaluate whether the principal findings were inflated by item content related to comprehension or personal relevance, the structural analyses were repeated using a narrower operationalization of community health information exposure. The narrower measure retained four items (CHE1–CHE4) that directly assessed frequency of message contact, diversity of community information sources, visibility of prevention messages, and attention to community health information. The three items primarily reflecting perceived comprehensibility, personal relevance, or subsequent cognitive engagement (CHE5–CHE7) were excluded from this sensitivity specification. The four-item measure retained satisfactory internal consistency (Cronbach's α = 0.842). Narrowing the exposure construct modestly attenuated its associations with the conceptually adjacent variables. The correlation between the narrower information-exposure measure and risk perception was *r* = 0.39 (*p* < 0.001), compared with *r* = 0.46 in the primary specification, while its correlation with health literacy decreased from *r* = 0.29 to *r* = 0.21 (*p* < 0.001). These reductions were consistent with the removal of item content related to comprehension and personal relevance. The structural model using the narrower exposure specification continued to demonstrate satisfactory fit (χ^2^/*df* = 1.938, CFI = 0.959, TLI = 0.953, IFI = 0.959, RMSEA = 0.037, SRMR = 0.044). Community health information exposure remained positively associated with risk perception (β = 0.351, *p* < 0.001) and chronic disease preventive behavior (β = 0.118, *p* = 0.004). Both coefficients were modestly smaller than their counterparts in the primary model (β = 0.417 and β = 0.143, respectively), indicating some attenuation after removal of the potentially overlapping item content without altering the direction or statistical interpretation of the associations. The chained indirect effect through risk perception and preventive self-efficacy also remained statistically supported. Based on 5,000 nonparametric bootstrap resamples, the indirect effect for the pathway community health information exposure → risk perception → preventive self-efficacy → chronic disease preventive behavior was 0.064 (95% bootstrap CI [0.039, 0.096]). Because the confidence interval did not include zero, the chained mediation pattern remained evident under the narrower exposure specification. Taken together, these sensitivity results suggest that the principal structural pattern was not attributable solely to the comprehension- or personal-relevance content included in the broader exposure measure. Nevertheless, the modest attenuation of the coefficients indicates that some shared semantic variance may have contributed to the magnitude of the primary associations. The direction and statistical interpretation of the principal findings were robust, whereas the absolute effect sizes should be interpreted with appropriate caution.

## 5 Discussion

### 5.1 Principal findings

The first major finding of this study is that community health information exposure had a significant positive association with chronic disease preventive behavior among middle-aged and older adults. This suggests that community communication should not be treated merely as a contextual background factor, but as an important starting point associated with residents' movement toward health action. For chronic conditions such as hypertension and diabetes, which require long-term management, community-based communication has the advantages of routine availability, accessibility, and repeated reinforcement, and can therefore continuously heighten attention to health management. At the same time, the coefficient of the direct path was not particularly large, indicating that information communication alone is not sufficient to explain the full range of behavioral change.

### 5.2 The chained pathway involving risk perception and preventive self-efficacy

The findings further indicate that the association between community health information exposure and chronic disease preventive behavior is statistically consistent with a continuous psychological process involving risk perception and preventive self-efficacy. Risk perception appears to function as an initiating link in the proposed model: community health information may help transform abstract knowledge about disease into a personally relevant sense of threat. Preventive self-efficacy then appears to act as a proximal link through which individuals who recognize risk develop the belief that they can take action and persist in doing so. Because the quantitative phase was cross-sectional, these interpretations should be read as pathway-consistent rather than as definitive evidence of causal sequencing. This chained pattern suggests that the practical value of health communication may depend not only on information arrival itself, but also on the extent to which risk is perceived and confidence is established. If communication content merely generates worry without strengthening action confidence, residents may remain in a “concerned but inactive” state. By contrast, when communication also provides understandable, actionable, and sustainable behavioral guidance, information is more likely to be translated into actual health practices.

### 5.3 The boundary role of health literacy

The present study found that health literacy significantly strengthened the association between community health information exposure and risk perception and was further associated with a stronger chained indirect path. This indicates that health literacy is not merely an auxiliary variable external to communication, but an important conditioning factor that may shape whether the associations linked to communication are stronger or weaker. Residents with higher health literacy are more able to understand medical terms, identify the most relevant points in health information, and transform those points into judgments about their own disease risk. Residents with lower health literacy, by contrast, may remain at the level of “having seen” or “having heard” the information without genuinely forming risk cognition or action readiness. These findings imply that improving health literacy among middle-aged and older adults is not only a long-term health promotion goal, but may also be an important strategy for increasing communication efficiency and promoting intervention equity in community-based chronic disease prevention (11, 18–20, 27).

### 5.4 Implications for public health practice

From the perspective of community public health intervention, simply increasing the frequency of communication is unlikely to guarantee behavioral formation. Community health education should not only emphasize disease risk but also provide more concrete and operable behavioral guidance, such as dietary substitution plans, demonstrations of monitoring procedures, regular exercise suggestions, family-doctor follow-up feedback, and small attainable goals. Such strategies may help translate abstract risk information into executable action plans. For groups with lower health literacy, reducing the threshold for information comprehension is especially important. Plain language, visual explanation, face-to-face demonstration, and continued reminders may improve information absorption and thereby increase the likelihood that residents will turn health knowledge into daily preventive behavior.

### 5.5 Strengths, limitations, and future directions

A principal strength of this study is the integration of community health information exposure, risk perception, preventive self-efficacy, health literacy, and chronic disease preventive behavior within a unified explanatory framework. The exploratory sequential mixed-methods design also enabled qualitative findings to inform construct specification and questionnaire development before the proposed relationships were examined quantitatively. The qualitative phase provided contextual insight into how middle-aged and older adults encounter, interpret, and respond to community health information, whereas the quantitative phase enabled the psychometric properties of the measures and the hypothesized structural relationships to be examined in a larger community-based sample. The additional discriminant-validity tests, moderated-mediation analysis, and sensitivity analyses further strengthened the assessment of the proposed model.

Several limitations should nevertheless be acknowledged. The quantitative phase used a cross-sectional design. Although the proposed structural model was theoretically ordered and statistically consistent with the hypothesized pathways, temporal precedence cannot be established from the present data. The findings should therefore be interpreted as associations that are consistent with the proposed mediation and moderation framework rather than as evidence of causal effects. Longitudinal, quasi-experimental, and intervention studies are needed to determine whether changes in information exposure precede subsequent changes in risk perception, preventive self-efficacy, and preventive behavior. Second, the principal constructs were measured using self-report data collected at a single time point. Residual common method bias and reporting bias therefore cannot be excluded despite the procedural precautions used during questionnaire administration (28). Chronic disease preventive behavior in particular represents self-reported practice rather than objectively verified behavioral performance. Future studies could combine questionnaire measures with community health records, objective physiological indicators, ecological momentary assessments, or wearable-device data.

Participants were recruited through site-based convenience sampling from community settings in Sichuan and Chongqing. The sample was therefore appropriate for examining the proposed relationships but should not be regarded as representative of the broader population of middle-aged and older adults. Complete recruitment-site identifiers were not retained for all respondents, preventing formal multilevel modeling of potential clustering by site. The estimated standard errors and confidence intervals should consequently be interpreted with this sampling structure in mind. Future research should use probability-based or stratified multisite sampling and retain complete site identifiers to permit explicit assessment of between-community heterogeneity. Eligibility required respondents to have encountered at least one source of community health information during the preceding 6 months. This criterion restricted the lower end of the exposure distribution and means that the findings primarily describe variation in exposure and engagement among residents who had already been reached by community health communication. The present study therefore cannot estimate the contrast between complete non-exposure and exposure. Future studies should include residents across the full range of community health information exposure. The conceptual boundary of community health information exposure warrants caution. The primary seven-item measure included not only message contact, source diversity, visibility, and attention but also perceived comprehensibility, personal applicability, and cognitive engagement. Some of these latter components are conceptually adjacent to health literacy and risk perception and may therefore contribute shared semantic variance. The HTMT analysis, alternative measurement-model comparisons, and narrower-exposure sensitivity analysis reduced concern that the principal structural findings were attributable entirely to this overlap. In particular, the principal associations and chained indirect pathway remained statistically supported when CHE5–CHE7 were excluded. Nevertheless, the attenuation observed under the narrower specification indicates that some overlap may have contributed to the magnitude of the primary coefficients. Future studies should distinguish more sharply among message exposure, message engagement, general health-literacy capacity, and subsequent risk appraisal, preferably using independently validated or multimethod measures. The project archive available for the present revision did not contain the complete original item-level response matrix and complete recruitment-site information. This limits the extent to which some item-level and site-level analyses can be independently reconstructed from the archived materials. Future mixed-methods studies should establish prospective reproducibility procedures that preserve the de-identified item-level dataset, data-cleaning records, complete analysis syntax, site identifiers where ethically permissible, and versioned statistical outputs.

### 5.6 Theoretical and policy implications

Theoretically, the present study extends the understanding of community health communication by moving beyond a linear “information dissemination–knowledge acquisition” account toward a more continuous “information dissemination–risk cognition–action belief–behavior implementation” framework. This explanatory mode may better match the observed formation logic of chronic disease preventive behavior in community public health practice. At the policy level, the present findings suggest that community health communication may benefit from moving beyond generalized publicity and one-way notification (6, 20, 21, 27). Instead, it could be combined with strategies for health literacy improvement, risk communication, and behavioral support. For example, family-doctor follow-up, health lectures, and community WeChat groups could include stratified content tailored to residents with different levels of health literacy in order to improve both comprehensibility and executability. These implications should nevertheless be treated as suggestive rather than as direct evidence of intervention effectiveness.

## 6 Conclusion

The present study examined the association between community health information exposure and chronic disease preventive behavior among middle-aged and older adults and tested a public health behavioral model that incorporated chained mediation and moderation. The findings showed that community health information exposure was associated with preventive behavior both directly and indirectly through risk perception and preventive self-efficacy, and that health literacy was associated with a stronger information–psychology–behavior pathway. The apparent effectiveness of community health communication may depend not simply on whether information reaches people, but also on whether risk is perceived, confidence is built, and action is supported. These findings provide a more complete explanatory framework for understanding the formation of chronic disease preventive behavior among middle-aged and older adults and offer cautious theoretical and practice-oriented implications for community health education, primary public health services, and precision intervention design.

## Data Availability

The raw data supporting the conclusions of this article will be made available by the authors, without undue reservation.
